# Engineering Tripartite Gene Editing Machinery for Highly Efficient Non-Viral Targeted Genome Integration

**DOI:** 10.21203/rs.3.rs-3365585/v1

**Published:** 2023-10-23

**Authors:** Hangu Nam, Keqiang Xie, Ishita Majumdar, Shaobo Yang, Jakob Starzyk, Danna Lee, Richard Shan, Jiahe Li, Hao Wu

**Affiliations:** 1. Department of Bioengineering, Northeastern University, Boston, MA 02115, United States; 2. Full Circles Therapeutics, INC. 625 Mount Auburn St., Ste. 105, Cambridge, MA 02138, United States; 3. Department of Biomedical Engineering, College of Engineering and School of Medicine, University of Michigan, Ann Arbor, MI 48109, United States

**Keywords:** Circular single stranded DNA, CRISPR/Cas9, homologous directed repair, CAR-T, genome integration

## Abstract

Non-viral DNA donor template has been widely used for targeted genomic integration by homologous recombination (HR). This process has become more efficient with RNA guided endonuclease editor system such as CRISPR/Cas9. Circular single stranded DNA (cssDNA) has been harnessed previously as a genome engineering catalyst (GATALYST) for efficient and safe targeted gene knock-in. Here we developed enGager, a system with enhanced GATALYST associated genome editor, comprising a set of novel genome editors in which the integration efficiency of a circular single-stranded (css) donor DNA is elevated by directly tethering of the cssDNA to a nuclear-localized Cas9 fused with ssDNA binding peptides. Improvements in site-directed genomic integration and expression of a knocked-in DNA encoding GFP were observed at multiple genomic loci in multiple cell lines. The enhancement of integration efficiency, compared to unfused Cas9 editors, ranges from 1.5- to more than 6-fold, with the enhancement most pronounced for transgenes of > 4Kb in length in primary cells. enGager-enhanced genome integration prefers ssDNA donors which, unlike traditional dsDNA donors, are not concatemerized or rearranged prior to and during integration Using an enGager fused to an optimized cssDNA binding peptide, exceptionally efficient, targeted integration of the chimeric antigen receptor (CAR) transgene was achieved in 33% of primary human T cells. Enhanced anti-tumor function of these CAR-T primary cells demonstrated the functional competence of the transgenes. The ‘tripartite editors with ssDNA optimized genome engineering’ (TESOGENASE^™^) systems help address the efficacy needs for therapeutic gene modification while avoiding the safety and payload size limitations of viral vectors currently used for CAR-T engineering.

## INTRODUCTION

The CRISPR-associated protein 9 (Cas9) is an effective and precise gene-editing enzyme that has revolutionized diverse fields of biotechnology, medical and agricultural research (Cong et al., 2013; Doudna and Charpentier, 2014; Hsu et al., 2014). Cas9 proteins coupled to a guide RNA find and induce site specific double-stranded breaks (DSB) into the genome for targeted gene deletion, repair or insertion in prokaryotes and eukaryotes (Cong et al., 2013; Gilbert et al., 2013; Mali et al., 2013; Qi et al., 2013). Variants of CRISPR/Cas9 tailored and optimized for a variety of site-specific genome editing/modification uses have been developed (Cong et al., 2013; Hilton et al., 2015; Komor et al., 2016; Liu et al., 2016). However, targeted insertion of a new DNA sequence into the genome remains challenging (Strecker et al., 2019). Conventional targeted gene knock-in (KI) is achieved by homologous recombination (HR) which is extremely inefficient, especially for longer sequences (Ding et al., 2019). Homology directed repair (HDR), is currently one of the most efficient ways to insert a DNA fragment ranging from a few bases to 2Kb, particularly when RNA-guided Cas9 introduced double-stranded breaks into the genome target (Roth et al., 2020, 2018; Zhang et al., 2017). But the efficiency of targeted insertion remains extremely low for > 4Kb large donor payloads (Mata López et al., 2020; Nishizono et al., 2020; Zhang et al., 2017). Non-targeted gene insertion remains an option mostly achieved with viral vectors and transposon/transposase system.

Therapeutic uses of gene insertion will benefit from methods that integrate donor DNAs of any size in predictable genomic locations. Successful treatment requires a high percentage of cells accurately engineered to provide a positive effect on the patient while avoiding the safety issues and random integration that have occurred for many viral vectors (Dever et al., 2016; Roth et al., 2018). Our prior studies showed that rearrangements and concatemerizations of the transgene prior to genomic insertion can be avoided by using circular single-stranded DNA (cssDNA) donors (Xie et al., 2022). Non-viral cssDNAs also reduce the cGAS-mediated cell toxicities imparted by dsDNAs and avoid the sometimes-life-threatening reactions to viral vectors that have plagued therapeutic adoption of virus-based vectors. The cssDNAs have been proven to be competent for Cas9-mediated, gRNA site-directed integration into the genome (Iyer et al., 2022; Xie et al., 2022). An added advantage is that cssDNAs of up to 20 kb can be readily grown in and purified using the recently described GATALYST vector that requires ~ 500 nt of vector sequence for replication in bacteria (Xie et al., 2022), compared to the >2 kb of vector sequence required for traditional phagemid vectors.

As HDR mediated genome integration is a highly spatially and temporally regulated process in nucleoplasm, the strategy to enhance the KI efficiency using DNA donor is 1) to efficiently deliver donor DNA into the nucleus and 2) to tether donor DNA onto the targeted genomic locus, both of which are critical steps to increase the active local concentration of the donor template to ensure effective homologous searching and pairing to complete the HDR process. This idea has been verified by multiple studies using different strategies. For instance, dsDNA donors have been coupled to Cas9 endonuclease by fusing Cas9 together with DNA binding proteins including transcription factors, recombinase subunits (such as Rad51, Rad52, POLD3) and the dominant negative form of 53BP1 to improve the efficiency of dsDNA knock-in (Gao et al., 2022; Jayavaradhan et al., 2019; Li et al., 2021; Lin-Shiao et al., 2022; Park et al., 2021; Rees et al., 2019; Reint et al., 2021; Shao et al., 2017, 2017; Tran et al., 2019). Other studies coupled the single or double stranded donor DNA to Cas9 in vitro using chemical modifications, such as DNA biotinylation for tethering to an avidin-conjugated Cas9 (ref) or 5’-triethylene glycol (TEG) modification or covalent conjugation of donor DNA with Cas9 endonuclease (Aird et al., 2018; Carlson-Stevermer et al., 2017; Ghanta et al., 2021; Ma et al., 2017; Savic et al., 2018). Other tethering strategies involve the creation of a sgRNA/donor DNA hybrid (Aiba et al., 2022; Nguyen et al., 2020; Shy et al., 2022; Simone et al., 2021). Overall, the majority of the strategies focus on dsDNA as donor templates whereas the cssDNA has proven to be a more efficient and safer donor DNA for targeted gene KI across multiple cell types to many targeted loci in the mammalian genome (Xie et al., 2022).

Here, we developed a set of enhanced GATALYST associated genome editors (enGager) by fusion of a nuclear localization single (NLS) peptide-tagged wild type Cas9 together with various single stranded DNA binding protein domains and peptides. These enGagers form a tripartite complex with sgRNA and cssDNA donors as an integrative site-specific, nucleus-targeted genome integration machinery. When applied for targeted genome integration with cssDNA donor templates to diverse genomic loci in various cell types, these enGagers outperform unfused canonical endonuclease editors by 1.5- to more than 6-fold. Cas9-cssDNA peptides and delivery methods optimized to achieve successful and functional, site-specific targeted integration in up to 33% of primary cells, dubbed the Tesogenase^™^ (tripartite editing with ssDNA optimized genome engineering nuclease) system, add a set of novel endonuclease into the gene-editing toolbox for potential cell and gene therapy development based on ssDNA mediated non-viral genome engineering.

## RESULTS

### Fusion of Cas9 with homologous recombination proteins enhance the efficiency of cssDNA mediated knock-in

**Fig. 1:**
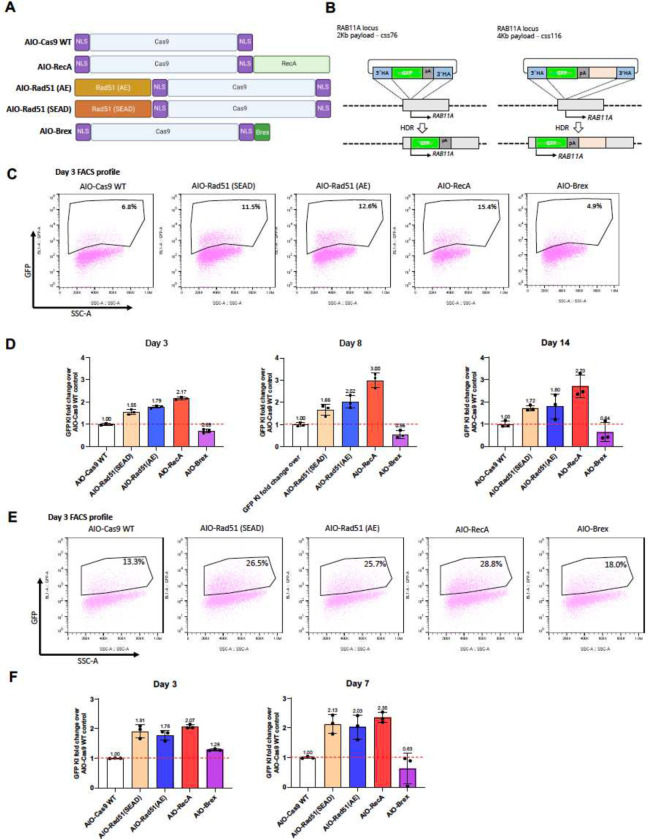
Fusion of Cas9 and homologous recombination proteins enhance the ssDNA mediated knock-in A, Schematic diagram of various Cas9-homologous recombination protein fusion constructs (enGagers) in all-in-one (AIO) plasmid format modified from Addgene plasmid #42230. Two nuclear localization signals were added to the N’ and C’-termini of the Cas9 protein. RecA is the bacteria homologous DNA repair protein and Rad51(AE)/Rad51(SEAD) are two mutant variants from eukaryotes. Brex is a 36 amino acid peptide reported to recruit Rad51 in mammalian cells. B, Schematic diagram of Knock in strategy of a 2Kb (left) and 4Kb (right) cssDNA donor template for RAB11A locus. C, representative FACS profiles with gating strategy showing % of GFP transgene cassette Knock in on RAB11 locus at day 3 post electroporation for various enGagers listed in A. D, Quantification of 2Kb GFP transgene cassette Knock in fold change of various enGagers as compared to Cas9 WT at day 3 (left), 8 (middle) and 14 (right) post electroporation. E, representative FACS profiles with gating strategy showing % of 4Kb GFP transgene cassette Knock in on RAB11 locus at day 3 post electroporation for various enGagers listed in A. F, Quantification of 4Kb GFP transgene cassette Knock in fold change of various enGagers as compared to Cas9 WT at day 3 (left), 7 (right) post electroporation. Note that Brex enGager does not enhance knock in efficiency. Rad51 mutants and RecA enGagers increase both small and large transgene cassette knock in by 1.57 to 3.04-fold. RecA enGager outperforms among the enGagers tested. Bars represent mean ± SD from 3 biological replicates.

We first evaluated the insertion of EGFP-coding sequence into the *RAB11A* genomic locus as our model for defining how much Cas9 modifications improve the efficiency of homology-directed, Cas9-dependent integration of a transgene (Fig. 1). A 2 kb cssDNA consists of a positive strand of 734 nucleotide long EGFP flanked by human genomic *RAB11A* sequences of 306 nt at the 5’ and 315 nt at the 3’, which can be targeted by a gRNA (Roth et al., 2018). The donor cssDNA was co-electroporated into human K562 lymphoblast cells with an all-in-one (AIO) plasmid expressing both the Cas9 enzyme and the gRNA that directs Cas9 to the *RAB11A* genomic site. Flow cytometry defines the percentage of cells in which the otherwise unexpressed EGFP codons are accurately inserted into a gene and expressed under the control of the endogenous *RAB11A* promoter (Roth et al., 2018). The Cas9 used was previously described to be targeted to the cell nucleus via fusion with two nuclear localization sequences (NLS) (Cong et al., 2013).

The NLS-Cas9 and derivatives are shown schematically in Figure 1A. The *RAB11A* genomic locus is shown in Figure 1B. Figures 1C and 1D show the percentage of green fluorescing cells detected by flow cytometry 3 days, 8 days and 14 days after electroporating the cssDNA and Cas9/gRNA plasmid into human K562 cells. Integration of the cssDNA catalyzed by NLS-Cas9 was compared with that supported by NLS-Cas9 fused to the *E. coli* RecA or the homologous human Rad51 DNA repair proteins previously shown to enhance Cas9-mediated integration (Chen et al., 2008; Lin et al., 2006; Shinohara et al., 1992; Su et al., 2016) (Fig 1); two mutant forms of Rad51 with enhanced recombigenic activity (AE and SEAD) (Rees et al., 2019) were used. NLS-Cas9 was also fused to a 36 amino acid (AA) peptide “Brex” (Ma et al., 2020) to recruit endogenous Rad51 recombinase to the NLS-Cas9 complex. Since both RecA and Rad51 also are ssDNA binding proteins, whereas the Brex peptide is not, the higher percentage of green fluorescence observed upon transfection with NLS-Cas9-Rad51 and -RecA fusions (11.5%-15.4%) compared to the NLS-Cas9-Brex fusion (4.9%) or to NLS-Cas9 (6.8%) provided a preliminary indication that Rad51/RecA fusion might improve the efficiency of recombination via their DNA binding properties; the hypothesis is that those DNA-binding proteins tether the cssDNA to the nuclear-localized NLS-Cas9 complex. Similar results also were obtained with a 4 kb cssDNA in which the GFP sequences and additional sequences were flanked by the same 5’ and 3’ *RAB11A* sequences surrounding the integration site (Figs 1B, E, F). The larger DNA insertions also were enhanced by the possible tethering of the cssDNA donor to NLS-Cas9 via ssDNA binding modules.

Knock-in enhancement by RecA and Rad51 persisted over time at 7/8 days and 14 days post-electroporation (Fig 1D, F) which is consistent with enduring expression following RecA/Rad51- enhanced integration. In general, enhancement by any of RecA or Rad51 averaged around 2-fold above that attained with unfused NLS-Cas9 across all studies/days.

### Engineering compact ‘enGagers’ in which NLS-Cas9 is fused with 20 aa ssDNA binding motifs

**Fig. 2:**
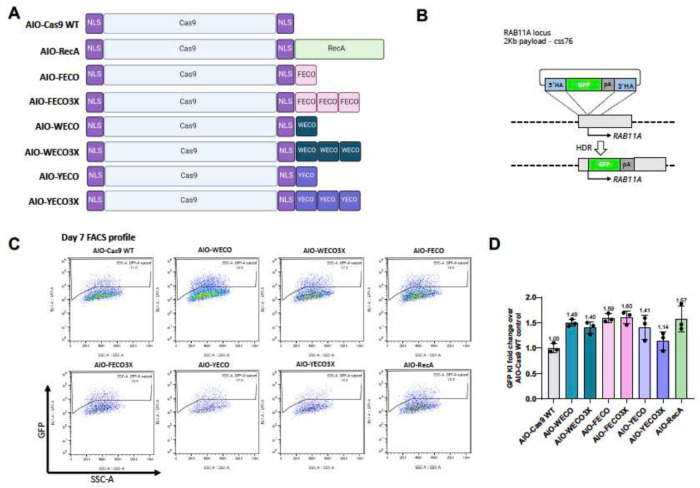
Identification of mini enGagers with Cas9 fused ssDNA binding motifs in K562 cells A, Schematic diagram of various Cas9-ssDNA binding motifs fusion constructs (enGagers) in all-in-one (AIO) plasmid format modified from Addgene plasmid #42230. Two nuclear localization signals were added to the N’ and C’-termini of the Cas9 protein. Cas9-RecA fusion construct was used as a positive control. FECO, WECO and YECO are 20 amino acid sequences previously identified as ssDNA binding motifs in various bacteria species of RecA(Voloshin et al., 1996), FECO3X, WECO3X and YECO3X are 3 tandem copies of the 20 aa peptides separated by multi-GS peptide linkers. B, Schematic diagram of Knock in strategy of a 2Kb cssDNA donor template for RAB11A locus. C, representative FACS profiles with gating strategy showing % of GFP transgene cassette Knock in on RAB11 locus at day 7 post electroporation for various small enGagers listed in A. D, Quantification of 2Kb GFP transgene cassette Knock in fold change of various enGagers as compared to Cas9 WT at day 7 post electroporation. Note that Cas9-FECO fusion performs similarly with Cas9-RecA fusion in cssDNA mediated transgene integration (1.59- vs 1.58-fold). EnGagers with 3X tandem ssDNA binding peptides do not further enhance knock in efficiency does not enhance knock in efficiency. Bars represent mean ± SD from 3 biological replicates.

We directly tested the hypothesis that enhancement of targeted cssDNA integration was permitted by the DNA-binding properties of RecA/Rad51. Several seminal biochemical studies on RecA family DNA recombinases identified an evolutionarily conserved ssDNA-binding L2 loop peptide that encompasses 20–24 amino acids with an extremely conserved central aromatic residue (Maraboeuf et al., 1995; Shinohara et al., 1992; Sugimoto, 2000; Sugimoto and Nakano, 1997; Voloshin et al., 1996). To test our hypothesis, the 20 amino acid RecA peptide sequences FECO, WECO and YECO (Voloshin et al., 1996) were first appended, as one or three tandem copies, to NLS-Cas9 linked by a Gly-Ser repeat polypeptide (Fig. 2A). When co-electroporated together with the 2 Kb cssDNA *RAB11A* GFP donor template into K562 cells (Fig. 2B), these newly designed NLS-Cas9-peptide fusions provided enhanced knock-in efficiencies similar to that of full length NLS-Cas9-RecA (p > 0.05). Except for the YECO3X construct, the fold enhancement ranged from 1.40 to 1.60 above that of NLS-Cas9, whereas the NLS-Cas9-RecA enhancement was 1.57-fold (Fig.2C,D). Adding multiple copies of the peptide motif did not further enhance the knock-in efficiency for this 2 Kb cssDNA *RAB11A* GFP donor payload. These data validated the hypothesis that just installing the sequence independent cssDNA binding capability to nuclease editors could effectively enhance homologous recombination-mediated gene insertion. These rationally designed NLS-Cas9-ssDNA binding peptides are referred to as ‘enGagers’(enhanced GATALYST associated genome editors). enGagers could be powerful editing tools for targeted genome integration where a donor DNA is needed.

### Optimization of SSB domains driven by SSB homologies

**Fig. 3:**
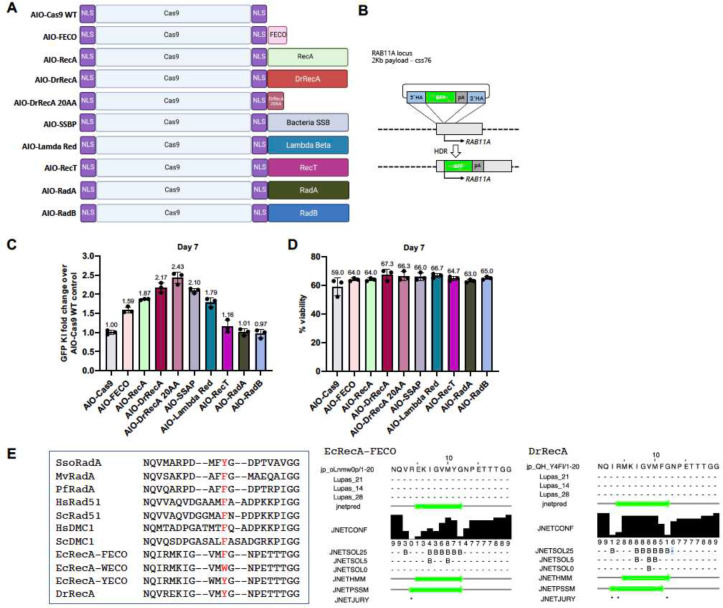
Identification of additional enGagers from Cas9-ssDNA binding module chimeras in K562 cells A, Schematic diagram of various Cas9-ssDNA binding protein and peptide fusion constructs (enGagers) in all-in-one (AIO) plasmid format modified from Addgene plasmid #42230. Two nuclear localization signals were added to the N’ and C’-termini of the Cas9 protein. Cas9-RecA and Cas9-FECO fusion constructs were used as positive controls. The fusion protein or peptides include DrRecA, 20 aa motif identified from DrRecA, SSAP, Lambda Red, RecT, RadA and RadB from Archaea. B, Schematic diagram of Knock in strategy of a 2Kb cssDNA donor template for RAB11A locus. C, Quantification of 2Kb GFP transgene cassette Knock in fold change of various enGagers as compared to Cas9 WT at day 7 post electroporation. Note that Cas9-DrRecA and Cas9-DrRecA20AA fusion has the highest performance in knock in with 2.17- and 2.43-fold as compared to Cas9 WT, respectively. D. Quantification of cell viability day 7 post electroporation. Bars represents mean ± SD from 3 biological replicates. E. Amino acid sequence alignment of 20AA of multiple E.coli RecA mutant variants and RecA from archaea and mammalian organism. Dr: *Deinococcus radiodurans*; Ec: *Escherichia coli; Sc: Saccharomyces cerevisiae; Hs: Homo sapiens; Pf: P. furiosus; Sso: S. solfatarcus.*

To engineer a larger collection of enGager constructs, we similarly tested a broader number of protein domains or peptides with ssDNA binding capability (Figs. 2A, 3A). We included the *Deinococcus Radiodurans* full length RecA homolog (DrRecA) and its 20 amino acid L2 peptide (Slade et al., 2009a; Zahradka et al., 2006), E.coli ssDNA binding protein (Meyer and Laine, 1990), Lambda Red subunit of bacteriophage recombineering protein (Mosberg et al., 2010), E.coli ssDNA annealing protein RecT (Noirot and Kolodner, 1998), and the Archaea recombinase homologs RadA and RadB (Seitz et al., 1998; Wardell et al., 2017). (Fig.3A&B). Fusions to NLS-Cas9 were screened using the 2 Kb cssDNA RAB11A GFP payload in K562 cells. Whereas fusions with RecT, RadA and RadB performed no better than Cas9 alone, all the other fusion constructs outperform Cas9 WT by 1.79- to 2.43-fold (Fig. 3C) without compromising the cell viability (Fig.3D). Interestingly, the enGager with a full-length DrRecA fusion stimulates *RAB11A* GFP KI by 2.17-fold and the homologous 20 amino acid L2 peptide from DrRecA further improves the KI efficiency level by 2.43-fold over that of WT Cas9. In comparison, FECO and RecA enGagers increase KI efficiency by 1.59- and 1.87-fold respectively. It is worth noting that *Deinococcus Radiodurans* is one of the most radiation-resistant bacteria known to date due to its exceptional DNA repair capability by its DNA recombinase DrRecA (Slade et al., 2009b; Zahradka et al., 2006). We reasoned that the L2 loop peptide from the whole RecA phylogeny can be a good candidate fusion peptide as a compact version of enGagers to enhance HDR mediated genome integration. We also highlighted a few RecA homologous L2 peptide sequences from archaea bacteria, E.coli and mammalian organism with a central aromatic amino acid (Fig.3E).

### Genome integration enhancement by enGager is ssDNA-dependent

**Fig. 4:**
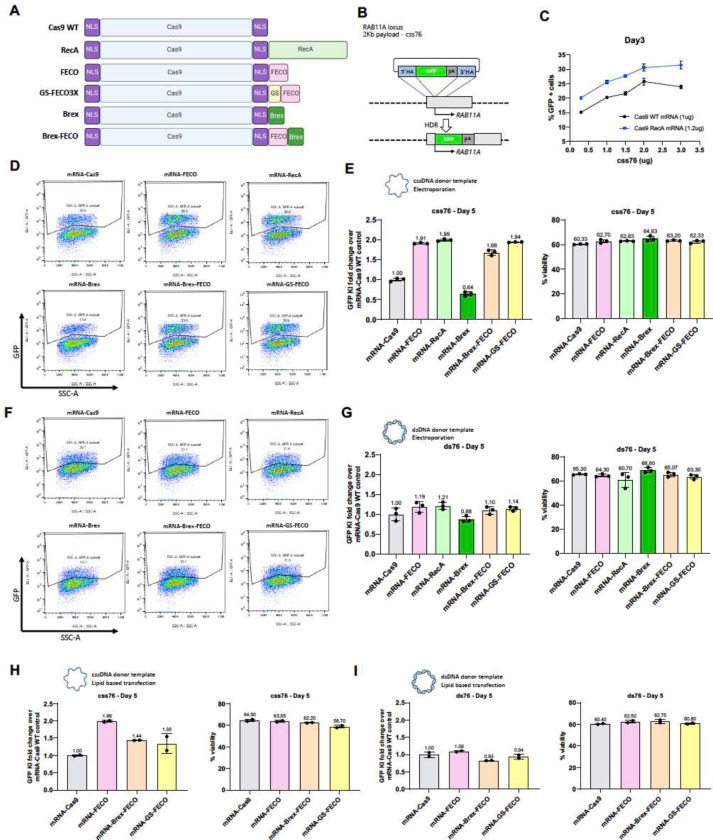
enGager mediated genome integration enhancement is ssDNA dependent A, Schematic diagram of various Cas9-ssDNA binding protein and peptide fusion constructs (enGagers) in mRNA form. Two nuclear localization signals were added to the N’ and C’-termini of the Cas9 protein. GS is a shortened poly-GS peptide linker. B, Schematic diagram of Knock in strategy of a 2Kb cssDNA donor template for RAB11A locus. C, Dose titration of cssDNA from 0.3, 1, 1.5, 2 and 3ug for 2Kb GFP transgene knock-in at day 3 post electroporation. Cas9-RecA mRNA enhance around 25–30% knock in efficiency than Cas9 WT mRNA at all the cssDNA dose tested in k562 cells. D, representative FACS profiles with gating strategy showing % of 2Kb GFP transgene cassette Knock-in on RAB11 locus by cssDNA donor at day 5 post electroporation for various enGagers listed in A in K562 cells. E. Quantification of 2Kb GFP transgene cassette Knock in fold change (left) and cell viability (right) of various enGagers as compared to Cas9 WT at day 5 post electroporation from D. F, representative FACS profiles with gating strategy showing % of 2Kb GFP transgene cassette Knock-in on RAB11 locus by dsDNA donor at day 5 post electroporation for various enGagers listed in A in K562 cells. E. Quantification of 2Kb GFP transgene cassette Knock in fold change (left) and cell viability (right) of various enGagers as compared to Cas9 WT at day 5 post electroporation from G. H, Quantification of 2Kb GFP transgene cassette Knock in fold change (left) and cell viability (right) of various enGagers mRNA as compared to Cas9 WT mRNA with cssDNA donor at 5 days post-delivery in HEK293 cells by lipofectamine 3000 transfection. I, Quantification of 2Kb GFP transgene cassette Knock in fold change (left) and cell viability (right) of various enGagers mRNA as compared to Cas9 WT mRNA with dsDNA donor at 5 days post-delivery in HEK293 cells by lipofectamine 3000 transfection. Bars represents mean ± SD from 3 biological replicates.

In the initial studies (Figs 1–3), the gRNAs were directly expressed from the same plasmid that delivered the NLS-Cas9-ssDNA binding motifs. Hereafter, except otherwise mentioned, we supplied the enGagers as in vitro transcribed mRNAs (Fig.4A). Using mRNAs has proved advantageous for reducing the cytotoxicity of transfected DNA and for enhancing delivery by using lipid nanoparticle technologies developed for vaccine development and gene therapy (Hou et al., 2021).

We initially validated the RecA enGager mediated enhancement of the 2Kb *RAB11A* GFP reporter integration in K562 cells by electroporation. When co-electroporated with various doses of cssDNA donor templates at 0.3, 1, 1.5, 2 to 3 ug per reaction, the full-length RecA enGager (at 1.2 ug mRNA per reaction) significantly increased the GFP KI efficiency compared to WT Cas9 mRNA provided at equimolar levels (Fig. 4B,C). Similar studies were conducted for the enGager mRNAs expressing the ssDNA binding and/or Brex recruitment peptides shown in Figure 4A. The results were similar to that observed with the enGagers delivered by AIO plasmids: the addition of an ssDNA binding peptide was as effective as full-length RecA at enhancing the percentage of cells expressing genomically integrated eGFP, with Brex peptides having no benefit (Fig. 4D, E).

As the RecA L2 peptides also bind dsDNA, albeit with much lower binding affinity compared to ssDNA (Voloshin et al., 1996), we sought to investigate if these enGagers can also facilitate integration of dsDNA donor templates. Using co-electroporation of mRNA enGagers/sgRNA and 1 ug of 2Kb *RAB11A* GFP donor template either in ssDNA or dsDNA form, we found that adding ssDNA binding motifs to Cas9 stimulated genomic integration only when the donor DNA was single-stranded (Figs. 4D, E) but not double-stranded (Figs. 4F, G). Similar dependence of ssDNA binding fusions on cssDNA donor templates were observed when the constructs were introduced into HEK293 cells by Lipofectamine 3000 (Figs. 4H, I).

We observed similar cellular viability of the K562 cells post-electroporation when the editing enzymes were provided as either RNA (Fig. 4E) or DNA (Fig. 3D) or when the donor DNA was provided as ssDNA or as dsDNA in either K562 (Figs. 4E, G) or HEK293 (Figs. 4H, I) cells.

### enGagers enhance ultra-large transgene integration on various genomic loci

**Fig. 5:**
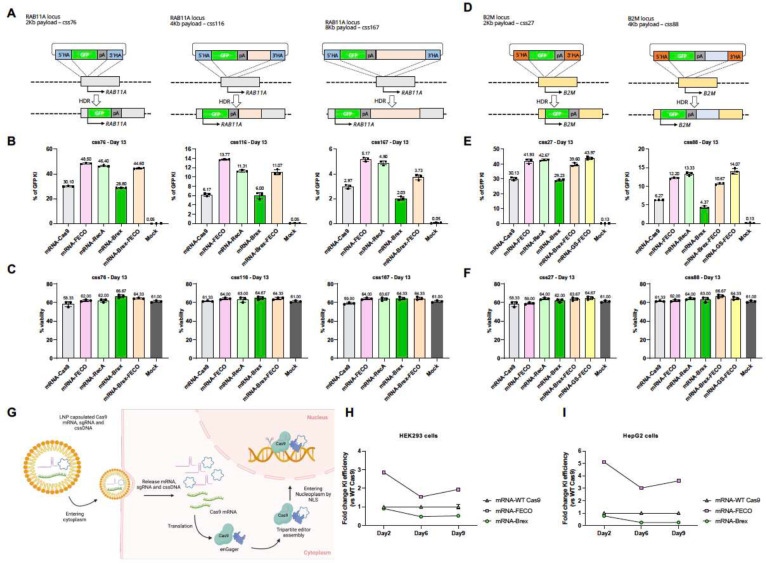
enGagers in mRNA form enhance genome integration on various locus and large payload A, Schematic diagram of Knock in strategy of 2Kb (css76), 4Kb (css116) and 8Kb (css167) cssDNA donor templates for RAB11A locus. B, Quantification of % of 2Kb (left), 4Kb (middle) and 8Kb (right) GFP transgene cassette Knock-in for various mRNA enGagers RAB11A day 13 post electroporation in K562 cells. C, Quantification of cell viability for 2Kb (left), 4Kb (middle) and 8Kb (right) GFP transgene cassette Knock-in for various mRNA enGagers on RAB11A locus day 13 post electroporation in K562 cells. D. Schematic diagram of Knock in strategy of 2Kb (css27), 4Kb (css88) cssDNA donor templates for B2M locus. E, Quantification of % of 2Kb (left) and 4Kb (right) GFP transgene cassette Knock-in for various mRNA enGagers on B2M locus day 13 post electroporation in K562 cells. F, Quantification of cell viability for 2Kb (left) and 4Kb (right) GFP transgene cassette Knock-in for various mRNA enGagers on B2M locus day 13 post electroporation in K562 cells. Bars represents mean ± SD from 3 biological replicates. G, Schematic diagram showing enGager mRNA/sgRNA/cssDNA delivery into cells using LNP formulation. Once the editor components were delivered into the cytoplasm, the enGager mRNA is translated into endonuclease protein which forms a complex with sgRNA and cssDNA donor template. The assembled tripartite editing machinery complex then can be effectively shuttled into the nucleoplasm and tether onto the target genomic locus for transgene integration. H & I, Quantification of GFP transgene knock-in on RAB11A locus in HEK293 cells (H) and HepG2 cells (I) by LNP delivery at day 2, day 6 and day 9 post-delivery. Data were compared to mRNA-WT Cas9. Bars represents mean ± SD from 2 biological replicates.

We next assessed the efficacy of enGagers more broadly on various genomic loci and for donor payload with various size, especially for 4 and 8Kb payloads that are at the packing limit for AAV6 and lentivirus, respectively. Utilizing highly purified mRNAs for expressing the FECO and RecA enGagers, we tested the efficiency of GFP transgene KI on two genomic loci with various length of payload. For *RAB11A* target locus, we designed 2Kb, 4Kb and 8Kb cssDNA payloads (Fig. 5A). For another clinically relevant immune cell therapy *B2M* locus (Ren et al., 2017), we designed 2Kb and 4Kb cssDNA payloads (Fig.5D). When mRNA enGager/sgRNA with cssDNA donor templates were co-electroporated to K562 cells, FECO and RecA enGagers increased the KI efficiency of 2Kb *RAB11A* GFP to 44.6–48.5%, while only 30.10% was achieved by WT NLS-Cas9. For the 4Kb payload, FECO and RecA enGagers increased the GFP transgene KI to 11.07–13.77% compared to 6. 17% and for 8Kb payload, to 3.73–5.17% from 2.97% (Fig.5B). Similarly, at the *B2M* locus, FECO and RecA enGagers increased the KI efficiency to 39.60–43.97% from 30.13% for the 2Kb GFP transgene, and to 10.67–14.07% from 6.27% for the 4Kb GFP transgene (Fig.5E). As a negative control, Cas9-Brex fusion did not enhance genomic integration efficiency of the cssDNA. Cell viability was not affected by 1 ug Cas9 or enGager mRNA (Fig.5C and 5F) as compared to Mock electroporation.

### Compatibility with lipid nanoparticle delivery

For whole animal gene modifications, lipid nanoparticles (LNP) have been successfully used to deliver Cas9 family editors for gene deletion, base editing and HDR based short gene insertion in rodent and non-human primates (Farbiak et al., 2021; Hou et al., 2021; Musunuru et al., 2021; Qiu et al., 2021; Zhang et al., 2020). In humans, Gillmore et al successfully used a specialized LNP formulation in a clinical trial with an in vivo gene-editing approach to reduce the serum level of transthyretin (TTR) by targeting the TTR gene as a therapeutic approach to treat Transthyretin amyloidosis (ATTR) (Gillmore et al., 2021). We evaluated if the FECO enGager and cssDNA donor template could be co-delivered with LNP to potentiate EGFP transgene integration efficiency in various cell types (Fig. 5G). When a commercially available LNP preparation was used to deliver the 2Kb GFP transgene cssDNA into HEK293T (Fig. 5H) or HepG2 (Fig. 5I) cells, FECO enGager demonstrated a persistent enhancement of a 2Kb GFP integration and expression at the *RAB11A* locus over multiple days following LNP delivery. These data demonstrated that the enGagers can be delivered in mRNA form by LNP to substantively improve genome integration efficiency and can potentially be applied for in vivo genome modification.

### Efficient CAR-T engineering with EnGagers

**Figure 6:**
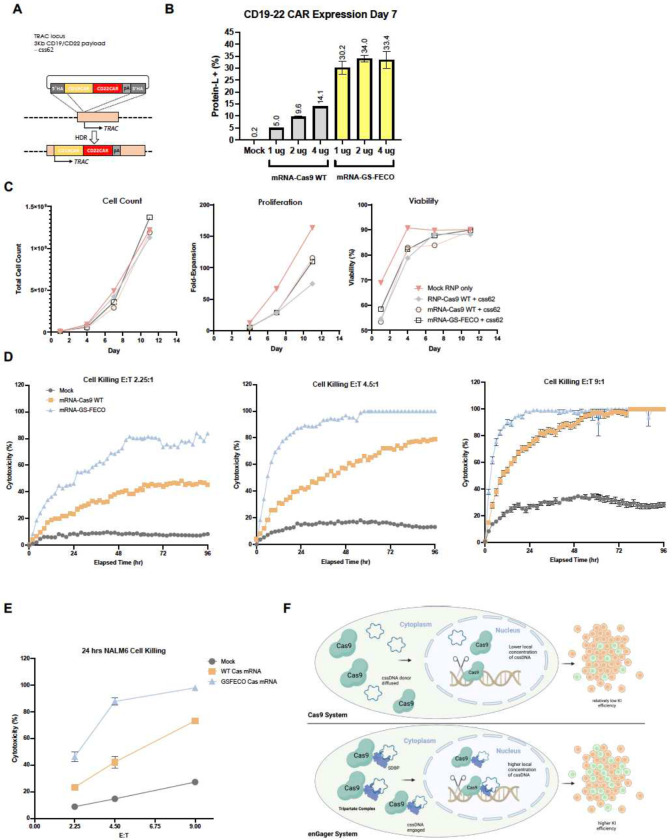
CAR-T engineering by enGager with superior efficiency than WT Cas9 A, Schematic diagram of Knock in strategy of 3Kb CD19/CD22 dual CAR (css62) cssDNA donor templates for TRAC locus in primary T cells. B, Quantification of % of CD19/CD22 CAR Knock-in using cssDNA donor analyzed by CD19 binder for various doses of Cas9 WT and Cas9-GS-FECO enGager mRNA at 1 ug, 2ug and 4ug. Data were collected at day 7 and day 11 post electroporation of primary T cells. Cas9-GS-FECO enGager achieves ~ 4- to 6-fold higher CAR-T engineering efficiency than Cas9 WT. C, Characterization of the engineered CAR-T cells or mock treated T cells for total cell count, cell proliferation fold change and cell viability over time. Bars represents mean ± SD from 2 biological replicates. D, NALM6 leukemia lymphocyte killing curve of unengineered T cells, CD19-CD22 dual CAR-T cells engineered with 2 ug of WT Cas9 mRNA and 2 ug of GS-FECO enGager mRNA over the course of 96 hrs. Effect (T cells): Target (NALM6 cells) are at 2.25:1 for left panel, 4.5:1 for middle panel and 9:1 for right panel. E, NALM6 cell killing function of CAR-T cells at 24 hrs for E:T ratio at 2.25:1, 4.5:1 and 9:1. F, Schematic diagram of engineered enGagers with single stranded DNA binding protein (SSBP) can recruit cssDNA donor template and form a tripartite editing machinery for efficient translocation of the entire editing complex from cytoplasm to nucleus. As a result, the donor DNA has higher effective local concentration in the nucleus for more efficient homologous directed genome integration. This process works more prominently with cssDNA.

Modifying a patient’s own T-cells in vitro followed by adoptive transfer back to the patient represents one of the most immediate applications for genome engineering for cellular therapy. We therefore examined the ability of the FECO enGager to engineer primary T cells by inserting a functional chimeric antigen receptor (CAR) transgene cassette within the T cell receptor alpha constant chain (*TRAC*) locus (Eyquem et al., 2017). We chose a ~ 3Kb CD19-CD22 dual CAR construct that has demonstrated anti-tumor function for potential treatment of patients with Acute Lymphoblastic Leukemia (ALL) and Non-Hodgkin’s lymphoma (NHL) (Fig. 6A and Fry et al., 2018). PE-conjugated Protein-L binder was used to analyze transgene expression as a marker for knock-in efficiency. When the CD19-CD22 dual CAR cssDNA donor template was delivered into human CD4+/CD8+ primary pan-T cells, the mRNA encoding one of the lead enGagers as evidenced in the K562 cell studies (GS-FECO) achieved 30.2% to 33.4% targeted CAR integration and expression at day 7 post-delivery, whereas WT NLS-Cas9 mRNA only achieved 5% to 14.1% of CAR KI over a range of enGager mRNA dosages (Fig 6B). Especially with the lower dose of mRNA editors preferred for studies involving patients, the GS-FECO enGager enhanced cssDNA-mediated CAR-T integration by >6 fold (Fig.6B). Importantly, GS-FECO enGager mediated enhancement of dual CAR-T engineering did not compromise T cell counts, proliferation and cell viability compared to WT Cas9 (Fig.6C). To test the anti-tumor function of the engineered CAR-T cells, we use the Incucyte live imaging approach to monitor the cell killing functions over the course of 94 hours. Cytotoxicity at NALM6 leukemia lymphocyte cells mediated by primary T-cells, modified by CD19-CD22 dual CAR integration under each enGager transfection condition, were determined at Effector (T cell): Target (NALM6 cells) ratios of 2.25:1, 4.5:1 and 9:1. In each instance, tumor cell killing by the GS-FECO enGager mRNA was more rapid than that of the NLS-Cas9 mRNA, presumably because of the much higher percentage of engineered CD19-CD22 dual CAR-T cells in the population. Importantly, at 9:1 E:T ratios, GS-FECO-modified T-cells enabled killing of 100% of NALM6 cells within 24 hrs and suppressed tumor cell regrowth. Overall, these studies demonstrated more effective and durable cancer cell killing function compared to WT Cas9 mRNA engineered dual CAR-T cells at every E:T ratio tested (Fig. 6D, E).

## DISCUSSION

In this study, we developed and characterized a set of enGager endonucleases by fusing Cas9 with various full-length single-stranded DNA binding protein modules or their corresponding miniaturized ssDNA-binding motifs of 20 amino acids. When applied for targeted genome integration with cssDNA donor templates to diverse genomic loci across multiple cell types, these enGagers outperformed conventional editors by up to several times for small and large transgene knock-in. Using one of the compact enGagers, we demonstrated sizeable chimeric antigen receptor (CAR) transgene integration in viral free manner in primary T cells with exceptional efficiency and anti-tumor cell function. As shown in Fig.6F schematic diagram, the engineered enGagers with single-stranded DNA binding protein (SSBP) can recruit cssDNA donor template and, together with a gRNA, for efficient editing of the target genome. The role that ssDNA binding plays in the hypothesized tethering of the donor DNA with the Cas9 endonuclease over the target site for enhancing gene modification is further suggested by the dependency of the integration on ssDNA (Fig. 4), which the appended motifs have a much stronger affinity for ssDNA than dsDNA (Voloshin et al., 1996). These newly designed enGager editors expanded the gene-editing toolbox for potential cell and gene therapeutic development based on ssDNA-mediated non-viral genome engineering.

Previously, it was challenging to achieve gene knock-in for >4kb payload especially for non-viral knock-in approaches. Although we observed integration efficiency to decrease with increasing donor DNA, we observed that improved efficiencies were realized by cssDNA tethering to the enGagers at all DNA sizes. Because the GATALYST cssDNAs can be produced with sizes of at least 20 kb (data not shown), whereas viral DNAs are more constrained in size, there is also the opportunity to considerably increase the size of the homologous sequences flanking the donor DNA to increase efficiencies with larger inserts. This flexibility makes gene knock-in improvement by enGager more attractive for many therapeutical applications that require large inserts to be functional. Additionally, the efficacy of enGagers was tested against different variables including multiple cell types, loci and payload sizes, and enGagers exhibited consistent improvement in knock-in efficiency. This improvement also includes an increase in efficiency for extremely large 8kb payload from ~3% to ~5%. Since many previous studies struggled to achieve meaningful knock-in for >4 kb payload, our finding may open new opportunities for precise gene therapy field. These new opportunities include multiple or large gene knock-in and long regulatory elements knock-in along with target gene. enGager also improves knock-in of smaller 2 kb payload, therefore the benefit of the enGager is not just limited to large payload. Also, unlike AAV viral mediated delivery, enGager delivers the donor cssDNA to the nucleus without the concerns of viral toxicity and unwanted integration while being relatively easy to manufacture.

One of the most critical findings of this study was that the 20aa single-stranded DNA binding motif of RecA showed comparable improvement relative to that of the full-length RecA protein. This observation underscores our hypothesis that the formation of tripartite complex rather than the recombination activity of RecA contributes to knock-in efficiency. We reason that the enGager platform can benefit from the 20aa motif over full-length proteins in several ways. First, the 20aa motif fusion enGagers are smaller than full protein fusion enGagers. Since increasing the size of Cas9 may sterically impede Cas9 function, minimizing the modification to 20aa motif is less likely to affect Cas9 editing function. Second, since the 20aa motif only has single-stranded DNA binding capacity, it is less likely to have the unwanted dsDNA-binding side effect that the full-length Rec-family protein have. Third, the extremely compact size of the 20aa motif fusion affords the opportunity for adding additional domains with other functions that may benefit Cas9 activity. While enGagers demonstrated significant improvement in gene knock-in efficiency, there are still some limitations of enGagers that calls for further studies. Further improvements in the ssDNA binding motifs added to the enGagers and/or altering the sizes of the homologous sequences surrounding the donor DNA remain to be followed-up to further enhance the extra-large transgene knock in.

## METHODS and MATERIALS

### Plasmid constructs

For all-in-one (AIO) plasmid construct design for various enGagers (Table 1), ssDNA binding protein and peptide sequences were synthesized and assembled into an AIO plasmid modified from Addgene plasmid #42230 by golden gate cloning. Complex fusion constructs were cloned using multi-piece DNA Gibson assembly approach. For mRNA constructs, various enGager coding sequences were cloned into a modified vector plasmid from pGEM^®^-4Z vector containing T7 promoter, 5’ UTR, Cas9 fusion sequence ORF 3’ UTR, bovine growth hormone polyadenylation signal (bGH polyA), and 64 poly adenine sequence (Promega). Table 1 listed the amino acid sequences of the enGager fusion protein and WT Cas9 endonuclease.

### mRNA production and purification

mRNA in this study was produced through in vitro transcription from plasmid templates. First plasmids containing T7 promoter, 5’ UTR, Cas9 fusion sequence ORF 3’ UTR, bovine growth hormone polyadenylation signal (bGH polyA), and 64 poly adenine sequence were cloned. To linearize the DNA, Spe1 restriction site was inserted at the end of the 64 poly adenines. After plasmids were produced, ~50μg of plasmids were digested using a restriction enzyme to cut the plasmids at the added restriction site for linearization. After 24 hours, the resulting linear DNA was cleaned through a DNA clean-up kit from zymo (Cat# D4029). After DNA cleanup, the linear DNA fragments were introduced to HiScribe^™^ T7 mRNA Kit with CleanCap^®^ Reagent AG (Cat# E2080S) from New England Biolabs. This kit used AG CleanCap^®^ Reagent from TriLink Biotechnologies and yielded ~ 100μg RNA per 1μg of linear DNA templates. For each reaction, 1μg of templates were mixed with 2μl 10x reaction buffer, 2μl of ATP (60mM), 2μl of GTP (50mM), 2μl of UTP (50mM), 2μl of CTP (50mM), 2μl of Cap Analog (40mM) and 2μl of RNA polymerase Mix. Then Nuclease-free water was added to the final volume of 20μl to the mixtures. The mixture was then incubated at 37C° for 2 hours. After 2 hours of in vitro transcription, the resulting mRNA was purified using RNA Clean & concentrator from Zymo (Cat # R1019). DNase was also used during the purification to remove residual DNA templates from the solutions. After in vitro transcriptions, each mRNA was analyzed using agarose gel analysis. 500ng of mRNA were diluted to 20ul of nuclease-free water then 40ul of 2 × RNA loading dye from ThermoFisher Scientific (Cat# R0641) was added. The mixture was then incubated at 80C° for 15 minutes. After incubation, the mRNA mixtures were immediately transferred to Ice and ran on the clean 1% agarose gel made from TAE buffer.

### LNP formulation and Delivery

1. HEK293T and HepG2 cells were plated on 24 well format day, a day prior to transfection in 500uls of antibiotics free and reduced serum (5%) DMEM media. 150k cells were plated / well of a 24 well plate
2. On the day of electroporation, cells were pretreated with 5uls of APoE (100X)
3. Using NanoAssemblr^®^ Spark^™^ system (Precision NanoSystems), 7 different formulations were prepared following the manufacturer protocol:
  - Formulation 1# Cas9 mRNA + RAB11A sgRNA + cssDNA
  - Formulation 2# FECO mRNA + RAB11A sgRNA + cssDNA
  - Formulation 3# Brex mRNA + RAB11A sgRNA + cssDNA
4. After preparation, formulations were diluted at 1:1 ratio.
5. LNP formulations were administered in 1:1 ratio in combination with css76 formulations
6. Cells were dissociated for FLO assays at day 2, day 6 and day 9 post-delivery.

### HEK293 lipofectamine transfection

For lipofectamine transfection of mRNA editors/sgRNA (mRNA cocktail) and DNA donors, 24 well plates are coated with PLO for 2hrs. Plates are washed 2X and dried before they were plated with cells. For each well 2X10^5^ cells were plated on Day 0 using 500 ul of complete DMEM media. On Day 1, 250uls of media was slowly replaced with equal volume of serum free and antibiotic free DMEM media. Both mRNA cocktail and DNA were prepared separately as per the conditions. To prepare the mRNA cocktail, individual mRNA construct (1ug/well) and Rab11A sgRNA (2.54ul/well from a stock of 80uM) were diluted in 1:1 ratio with lipofectamine 3000 and incubated on ice for 10–15 minutes; for DNA prep, both single stranded and double stranded DNAs were packaged separately. For both, concentration of 1ug/well was used. Respective DNA was mixed with P3000 reagent (1ug/well) and the whole mix was diluted with lipofectamine 3000 in 1:1 dilution. The DNA cocktail was also incubated at RT for 10–15 mins. After 15 mins of incubation, mRNA cocktail and DNA cocktail were added in 1:1 ratio as per the plate map. Cells were then allowed to sit for 48 hours after which they were transferred from 24 well plate format to 6 well plate with 1ml media. FLO analysis was performed on D5, D9 and D14 to check on the knock in efficiency of the constructs.

### Generation of circular single-stranded DNA

Donor template sequences (transgene sequence flanked with 5’ and 3’ homology arms at 300–500 nt in length) are constructed as dsDNA and placed into phagemid vector by Golden Gate Assembly. An XL1-Blue E. Coli Strain was co-transformed with the M13 helper plasmid and phagemid containing donor template sequences and selected in agar plate with kanamycin (50 μg/mL), carbenicillin (100 μg/mL). Single colony was selected and grown for ~24 hours (37 °C, 225 rpm) in 250 mL 2xYT media (1.6% tryptone, 1% yeast extract, 0.25% NaCl) to reach OD600 between 2.5–3.0. The bacteria were pelleted by centrifugation and the phage particles in the supernatant were precipitated by PEG-8000 by centrifugation, washed and lysed in 20 mM MOPS., 1M Guanidine-HCl and 2% Triton X-100. The cssDNA released from phage were then extracted with NucleoBond Xtra Midi EF kit (Macherey-Nagel) following the manufacturer’s instructions. The concentration of cssDNA is determined by Nanodrop. Ratios of Absorbance (A260 nm/280 nm and 260nm/230nm) will reflect consistent purity (1.8 and > 2, respectively) from serial preps. Recombinant cssDNA is verified by DNA sequencing using custom-designed staggered sequencing primers for complete coverage.

### Cell culture

K562 cells (ATCC, CCL-243) were maintained in RPMI-1640 media with 10% FBS and 1% penicillin and streptomycin. HEK293T (ATCC) cells were cultured in Dulbecco’s modified Eagle’s medium supplemented with 10% fetal bovine serum and 1% penicillin and streptomycin (Gibco). HepG2 cells (HB-8065^™^) were cultured in ATCC-formulated Eagle’s Minimum Essential Medium (Catalog No. 302003) supplemented with 10% FBS. iPSC (Thermo Fisher) was cultured in StemFlex (Thermo Fisher) media in vitronectin-coated flask. iPSC colonies were checked regularly and passaged using ReLeSR (StemCell Technologies) every 3–4 days of culture. iPSCs were ready for electroporation after 2–3 passages. T cells were cultured and expanded in TexMACS Medium (Miltenyi) supplemented with 200 IU/mL Human IL-2 IS (Miltenyi). T cells were activated for 2 days with T Cell TransAct (Miltenyi) before electroporation. All cells were maintained in a humidified incubator at 37 °C and 5 % CO2, unless otherwise specified. Cell count viability were determined using Via2-Cassette in NucleoCounter^®^ NC-202 (ChemoMetec) on specified days after engineering.

### Electroporation of Cas9 ribonucleoprotein, AIO plasmid and mRNA/sgRNA complex with DNA donor

All K562, HEK293T, iPSC and T cell electroporation were performed using the Amaxa^™^ 96-well Shuttle^™^ with the 4D Nucleofector (Lonza). Cas9 nucleases and sgRNAs were precomplexed in supplemented Nucleofector^®^ Solution for 20 min at room temperature and the RNP solution was made up to a final volume of 2.5 μL (10X). For electroporating K562 cells, SF Cell Line 4D-Nucleofector^™^ Kit and 250,000–500,000 cells per reaction were used with program FF-120. For electroporating HEK293T cells, SF Cell Line 4D-Nucleofector^™^ Kit and 200,000–300,000 cells per reaction were used with program FS-100. For electroporating iPSC cells, 100,000 cells per reaction were used with P3 Primary Cell 4D-Nucleofector^™^ Kit and program CA-137. For electroporating primary T cells, 2×10^6^ cells per reaction were used with P3 Primary Cell 4D-Nucleofector^™^ Kit and program EO-115. Indicated amount of HDR donor template (cssDNA or dsDNA) were co-electroporation with RNP. For mRNA or AIO plasmid enGager electroporation, 1 ug-1.5ug of mRNA or DNA were electroporated together with indicated amount of HDR donor templates, with the same electroporation parameters used for RNP electroporation. After electroporation, cells were incubated in humidified 32°C incubator with 5 % CO2 for 12–24 hours followed up transferring to 37°C incubator for additional days.

### Flow Cytometry analysis

All flow cytometry was performed on an Attune NxT flow cytometer with a 96-well autosampler (ThermoFisher Scientific). Unless otherwise indicated, cells were collected 3–14 days post electroporation, resuspended in fluorescence-activated cell sorting (FACS) buffer (2% BSA in PBS) and stained with 7-AAD (BioLegend) as a dye for cell viability assay, and the indicated cell-surface marker, such as PE labelled Protein L (ACROBiosystems), or FITC-Labeled Human CD19 (20–291) Protein, Fc Tag (ACROBiosystems). To obtain comparable live cell counts between conditions, events were recorded from an equivalent fixed volume for all samples. Data analysis was performed using FlowJo_v10.8.0_CL software with exclusion of subcellular debris, singlet gating and live/dead staining. Data analysis is performed using FlowJo_v10.8.0_CL software. Data were plotted using Prism Graphpad.

### CAR-T cell engineering

Primary T cells were isolated and enriched from Leukopak using Pan T Cell MicroBead Cocktail with MultiMCAS Cell24 Separator Plus (Miltenyi), CD4+ and CD8+ pan T cells were cryopreserved for later use. T cells were cultured and expanded in TexMACS Medium (Miltenyi) supplemented with 200 IU/mL Human IL-2 IS (Miltenyi). T cells were activated for 2 days with T Cell TransAct (Miltenyi) before electroporation. 48 h after initiating T-cell initiation and activation, T cells were electroporated using Amaxa^™^ 96-well Shuttle^™^ in 4D Nucleofector. 2×10^6^ cells were mixed with 25 pmol of Cas9 WT protein or 1 ug of enGager mRNA and 50 pmol of gRNA into each well. For cssDNA engineered cells, 2 μg of cssDNA encoding bi-specific CD19×CD22 CAR (Fry et al., 2018) was electroporated with RNP or mRNA/sgRNA cocktail targeting TRAC locus. Following electroporation, cells were diluted into culture medium in the presence of T Cell TransAct with 1 μM DNA-PK inhibitor M-3814, and incubated at 32 °C, 5% CO2 for 24 hours. Cells were then washed and subsequently transferred into G-Rex 24 Multi-Well Cell Culture Plate (Wilson Wolf) in standard culture conditions at 37 °C, 5% CO2 in IL-2 supplemented TexMACS medium and replenished every 3–4 days. CAR expressions were determined using PE labelled Protein L (ACROBiosystems), or FITC-Labeled Human CD19 (20–291) Protein, Fc Tag (ACROBiosystems).

## Highlights

- Improvement in targeted knock-in efficacy using ‘enGager’, a Cas9 nuclease fused with ssDNA binding peptides
- Optimization of ssDNA binding peptides for high efficiency enGager-mediated integration
- Highly efficient functional CAR-T cell engineering by enGager mRNA and ssDNA
- High efficiency enGager/cssDNA/gRNA systems have been dubbed TESOGENASE^™^ for tripartite editors with ssDNA optimized genome engineering.
